# *Notice to Readers*: Online Manuscript Submission System Now Available for *MMWR* Serial Publications

**DOI:** 10.15585/mmwr.mm6701a9

**Published:** 2018-01-12

The *MMWR* Serial Publications are now using ScholarOne Manuscripts, an online system for manuscript submissions. This system provides comprehensive workflow management and streamlines the submission process for *Recommendations and Reports*, *Surveillance Summaries*, and *Supplements*.

ScholarOne Manuscripts allows manuscripts to be transmitted electronically and makes manuscript files accessible to editors through the submission site. All manuscripts for the Serial Publications must be submitted through ScholarOne Manuscripts at https://mc.manuscriptcentral.com/mmwr-sp. Additional information on how to submit through ScholarOne Manuscripts is available at https://www.cdc.gov/mmwr/serial_submissions.html.

